# Non-invasive prediction of blastocyst implantation, ongoing pregnancy and live birth, by mass spectrometry lipid fingerprinting

**DOI:** 10.5935/1518-0557.20160044

**Published:** 2016

**Authors:** Edson Borges Jr., Daniela P.A.F. Braga, Amanda Souza Setti, Daniela A. Montanni, Elaine Cristina Cabral, Marcos N. Eberlin, Edson G. Lo Turco, Assumpto Iaconelli Jr

**Affiliations:** 1 Fertility Medical Group - São Paulo/SP - Brazil; 2 Instituto Sapientiae - Centro de Estudos e Pesquisa em Reprodução Assistida - São Paulo/SP - Brazil; 3 Disciplina de Urologia, Departamento de Cirurgia, Setor de Reprodução Humana - UNIFESP/SP - Brazil; 4 Laboratório ThoMSon de Espectrometria de Massas - Instituto de Química - UNICAMP

**Keywords:** Lipidomic, mass spectrometry, fingerprinting, implantation

## Abstract

**Objective:**

To identify lipid markers of blastocyst implantation and ongoing pregnancy by day three culture medium mass spectrometry (MS) fingerprinting.

**Methods:**

For this study, 33 culture media samples were harvested on day three, from 22 patients undergoing day five embryo transfers. All embryos achieved the blastocyst stage and were split into groups based on their implantation (Negative Implantation, n= 14 and Positive Implantation, n= 19). The positive implantation cycles resulted in successful ongoing pregnancies. The lipid extraction was performed by the Bligh-Dyer protocol and mass spectra were obtained with a direct infusion into a Q-Tof mass spectrometer. The data obtained was analyzed by Principal Component Analysis (PCA) and Partial Least Square Discrimination Analysis (PLS-DA). The statistical analysis was performed using the Metabo-Analyst 2.0.

**Results:**

The variable importance in the projection (VIP) plot of the PLS-DA provided a list of four ions, in the positive mode, with an area under the curve (AUC) of 73.5%; and eight ions, in the negative mode, with and AUC of 72.0%. For both positive and negative modes, possible biomarkers for the negative implantation were identified by the lipidmaps: phosphoethanolamine, dicarboxylic acids, glycerophosphoglycerol, glycerophosphocholine, glicerophosphoinositol, phosphoethanolamine and unsaturated fat acids. The other ions were not identified. These lipids are involved in the GPI anchor biosynthesis and synthesis of lycerophospholipids and phosphate inositol.

**Conclusion:**

MS fingerprinting is useful to identify blastocysts that fail to implant, and therefore this technique could be incorporated into the laboratory routine, adjunct to morphology evaluation to identify embryos that should not be transferred.

## INTRODUCTION

In vitro fertilization (IVF) success rates have been remarkably improved since the first successful birth in 1978 (Steptoe & Edwards, 1978). However, its efficiency, measured as live birth rate, is usually well below 50%.

This low efficiency contributes to the practice of multiple embryo transfer, which frequently leads to multiple pregnancies (Pandian *et al*., 2009; Setti & Bulletti, 2011). In vitro fertilization has been associated with a 30-fold increase in multiple pregnancies, compared with the rate of spontaneous twin pregnancies (ACOG 2005) and it is associated with a broad range of negative consequences for both the mother and the fetuses (ESHRE Capri Workshop Group, 2014).

Indeed, the need to decrease assisted reproduction-induced iatrogenic multiple pregnancies has become a health, economic, and legal issue in several countries (Adashi *et al*., 2003). The most effective approach to minimize the risk of multiple pregnancies is single-embryo transfer (SET). Nevertheless, there are concerns, that the use of only one embryo can reduce success rates (Grady *et al*., 2012; Steinberg *et al*., 2013). Therefore, successful implementation of SET depends on the ability to select the most viable embryo from a cohort, which remains a challenge despite the current use of numerous scoring systems

Prolonging the embryo culture period enables a better selection of embryos for transfer, because laboratory assessment is performed after the embryonic genome has begun to be expressed. However, due to the continuing inability to predict which blastocyst presents the higher implantation potential, the development of reliable and non-invasive methods of embryo evaluation is crucial.

Non-invasive approaches for embryonic development potential assessment have the advantages of increasing the knowledge regarding embryo physiology; therefore, enabling the development of methods to predict developmental competence and viability (Hamel *et al*., 2008). These approaches include genomic and proteomic profiling, analytical evaluation of the embryonic metabolome (Botros *et al*., 2008; Bromer and Seli 2008; Katz-Jaffe *et al*., 2009, Aydiner *et al*., 2010; Ferreira *et al.*, 2010; Seli *et al*., 2010; Cortezzi *et al*., 2011), and most recently: lipidomic profiling (Braga *et al*., 2015).

Modern approaches for lipidomic analysis are dominated by mass spectrometry (MS) (Want et al. 2005). The novel MS-based lipidomics methods enable the study of intact lipid molecular species from very small amounts of samples and such methods, due to their wide dynamic range, enable quantitative or relative determination of compounds across a broad range of concentrations (Schwudke *et al*., 2006).

Therefore, the goal for the present study is to identify lipid markers of blastocyst implantation and ongoing pregnancy by day three culture medium MS fingerprinting.

## MATERIALS AND METHODS

### Experimental Design

For this study, 33 culture media samples were harvested on day three from 22 patients undergoing day five embryo transfers. All embryos achieved the blastocyst stage and were split into groups based on their implantation (Negative Implantation, n= 14 and Positive Implantation, n= 19). Embryo secretomes were analyzed by MS.

Patients in the positive implantation group presented 100% implantation and positive implantation cycles resulted in successful ongoing pregnancies and seven successful live births, so far.

Patients signed an informed consent form, in which they agreed to share the outcomes of their cycles for research purposes. The local institutional review board approved the study (CEP: 1095/2015).

### Controlled Ovarian Stimulation and Oocyte Retrieval

Controlled ovarian stimulation was achieved by using recombinant FSH (Gonal-F; Serono, Geneva, Switzerland), at a daily dose, starting on day three of the cycle. Pituitary blockage was performed by using a GnRH antagonist (Cetrotide, Serono, Geneva, Switzerland), starting when at least one follicle ≥14 mm was visualized.

Follicular growth was monitored using transvaginal ultrasound examination starting on day four of the gonadotropin administration. When adequate follicular growth and serum 17β estradiol levels were seen, recombinant hCG (Ovidrel; Serono, Geneva, Switzerland) was administered to trigger the final follicular maturation. The oocytes were collected 35 hours after hCG administration through transvaginal ultrasound ovum pick-up.

### Preparation of Oocytes and intracytoplasmic sperm injection

Retrieved oocytes were maintained in culture medium for 5 hours. Surrounding cumulus cells were removed and oocytes were checked for oocyte maturation, and those which had released the first polar body (metaphase II oocytes - MII) were considered mature and used for Intracytoplasmic sperm injection (ICSI), which was performed using the technique described by Palermo *---*(1992).

### Fertilization and Embryo Quality Assessments and Embryo Transfer

Approximately 18h after ICSI, fertilization was confirmed by the presence of two pronuclei and the extrusion of the second polar body. Subsequently, embryos were transferred to new drops of culture medium to be individually cultured for 48 hours. On day three, the culture media was refreshed and spent culture media was collected and stored at -20ºc. The embryos were transferred to another dish and cultured until day five when embryo transfer was performed.

### Sample Preparation and Mass Spectrometry

Subsequent to the confirmation of implantation, the culture medium samples were divided according to their implantation outcomes.

The lipids from culture medium were individually extracted using the Bligh and Dyer method (Bligh & Dyer 1959), dried and diluted in 400 µL of MeOH.

Mass spectra were obtained with a direct infusion of both the negative and positive ion modes into a Q-Tof mass spectrometer (LC-MS, Agilent 6550 iFunnel Q-TOF) equipped with an automated injector.

### Data Analysis

The data obtained was analyzed by Principal Component Analysis (PCA) and Partial Least Square Discrimination Analysis (PLS-DA), combined with variable influence in the projection (VIP) scores, to identify potential biomarkers of blastocyst implantation and ongoing pregnancy. Statistical analysis was performed using the Metabo-Analyst 3.0.

## RESULTS

**Figure 1:**
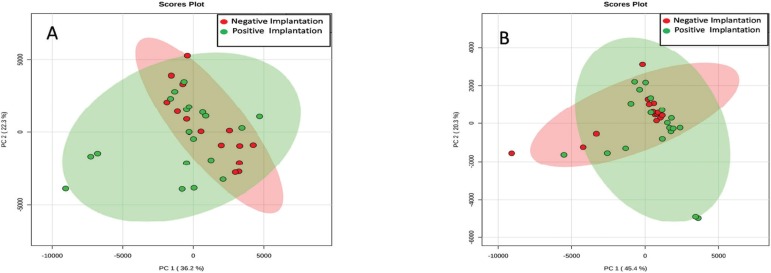
PCA plot of the scores for samples from the Positive and Negative implantation groups for the positive (A) and negative (B) modes.

The PCA analysis was performed to identify chemical differences between the Negative and Positive Implantation groups. Figure 1 shows the graphics of principal components (PC1 versus PC2) on the positive (Figure 1A) and negative (Figure 1B) modes. On the positive mode we noticed an increased difference for lipid characteristics between the negative and positive implantation groups, when compared with the negative mode.

Moreover, we could note a better clustering in the negative implantation group for both, positive and negative modes.

**Figure 2:**
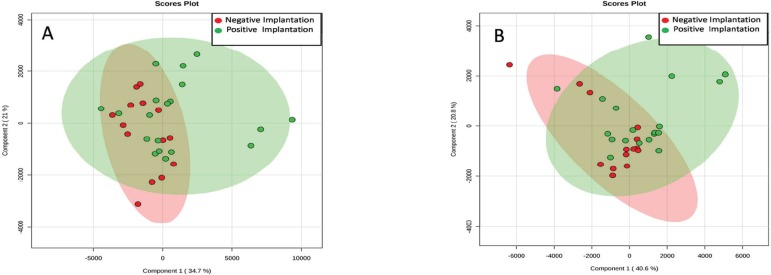
PLS-DA plot of the scores for samples from the Positive and Negative implantation groups for the positive (A) and negative (B) modes.

The PLS-DA was applied to evaluate differences in the lipidomic profile between the groups and to identify possible biomarkers of blastocyst implantation. Graphics on positive (Figure 2A) and negative (Figure 2B) modes showed satisfactory separations between the Positive and Negative Implantation groups.

**Figure 3:**
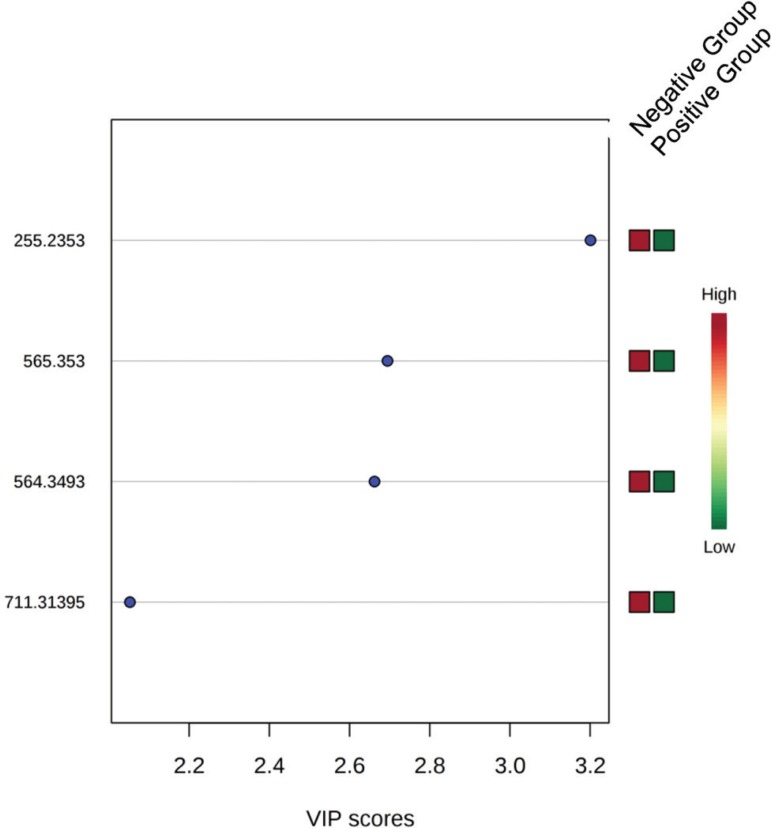
List of ions provided by the variable importance projection (VIP) scores of the PLS-DA model for the prediction of embryo implantation in the positive mode.

**Figure 4:**
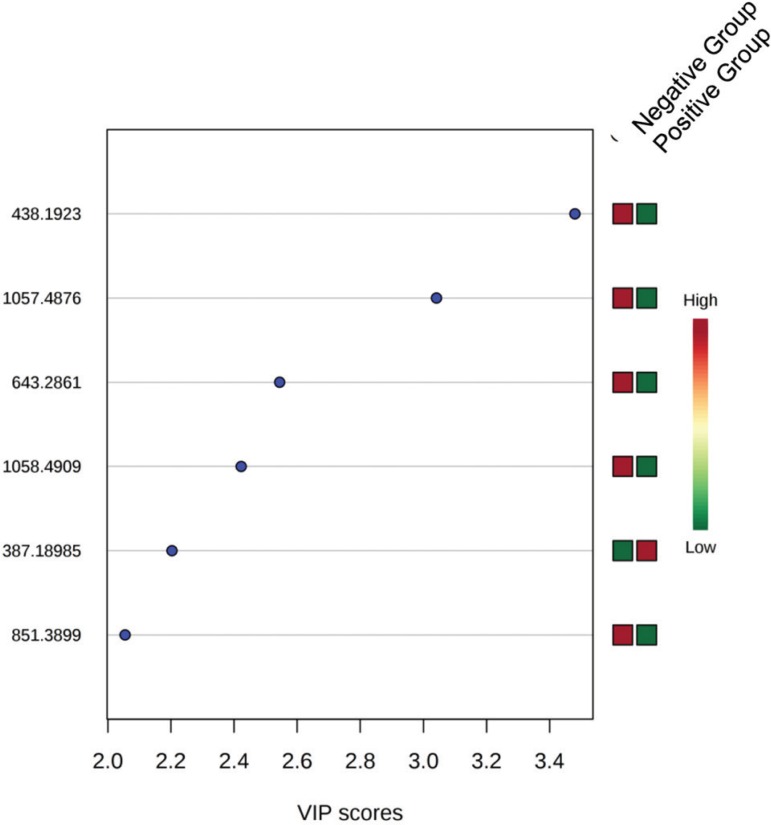
List of ions provided by the variable importance projection (VIP) scores of the PLS-DA mode for the prediction of embryo implantation in the negative mode.

The variable importance in the projection (VIP) plot of the PLS-DA provided a list of four ions, in the positive mode (Figure 3), with an area under the curve (AUC) of 73.5% and six ions, in the negative mode (Figure 4), with and AUC of 72.0%. For both positive and negative modes, possible biomarkers for the negative implantation were identified by the lipidmaps: phosphoethanolamine, dicarboxylic acids, glycerophosphoglycerol, glycerophosphocholine, glicerophosphoinositol, phosphoethanolamine and unsaturated fatty acids. The other ions were not identified. These lipids are involved in the GPI anchor biosynthesis and synthesis of glycerophospholipids and phosphate inositol.

## DISCUSSION

A key step in assisted reproduction is to identify the embryo(s) that are most likely to result in a pregnancy. In the present study, possible lipid biomarkers for blastocyst implantation fail were identified.

Non-invasive determinants of embryo viability are limited, and therefore current embryo assessment strategies involve embryo morphology, which is the easiest way to predict viability. However, the majority of studies suggest that the morphology of embryos with high-quality morphological appearance is insufficient for predicting a successful implantation (Katz-Jaffe *et al*., 2009; Assou *et al*., 2011; Mastenbroek *et al*., 2011). This method is highly subjective (Paternot *et al.,* 2009; Santos-Filho *et al*., 2010), and the correlation of morphological parameters and embryo implantation potential is unclear (Kovalevsky & Patrizio, 2005). Furthermore, morphologically normal embryos can be genetically abnormal because a significant proportion of aneuploid embryos can achieve the highest morphologic scores (Singh & Sinclair, 2007; Alfarawati *et al*., 2011; Assou *et al*., 2011).

Within this context, many metabolic parameters for developing embryos have been studied using a variety of non-invasive methods (Sakkas & Gardner, 2005). These studies demonstrate an underlying metabolic difference between the embryos that result in a pregnancy and those that do not, forming the basis of a metabolomic approach for assessing embryo viability.

More recently, studies have suggested that embryos with positive and negative implantation outcome alter their environment differently, which is reflected in the surrounding metabolites (Scott *et al*., 2008; Seli *et al.,* 2008; Vergouw et al., 2008; Seli *et al*., 2010; Ahlström *et al.*, 2011; Sfontouris *et al*., 2013).

In a previous study of our group, MS associated with the PLS-DA mode has been successfully employed for embryo viability prediction (Cortezzi *et al*., 2013). In this study, MS fingerprinting was used to predict human embryo implantation potential and the metabolomic profile was achieved.

For the present study, we specifically assessed the lipidomic profile. Although having been an intensive area of research already in the 1960s, lipid research has recently gained prominence with the emergence of lipidomics (Han & Gross, 2005; Oresic *et al.*, 2008). Lipidomics can be defined as the large-scale study of lipid species and their related networks and metabolic pathways that exist in cells or any other biologic system. Lipids have highly diverse functions other than cellular membrane structure and energy storage. It plays an important role in diverse biologic functions (Loizides-Mangold, 2013).

The present study was able to identify possible lipid involvement in cellular metabolism in culture media samples of non-implanted embryos. It could be argued that embryos who fail to implant may have used the cellular machinery to prevent apoptosis to exhaustion.

So far, the diagnostic power of such patterns is not completely conclusive since embryo implantation into the endometrium does not depend exclusively on proper embryo development, it also depends on other critical events, which include the acquisition of a receptive endometrium, and the appropriate dialogue between maternal and embryonic tissues (Dominguez *et al*., 2003).

Nevertheless, this technology seems to provide a fast, reliable, and noninvasive prediction tool to help the selection of the best embryo to be transferred, and should be used as an adjunct to morphological evaluation, thus minimizing the risks of the undesirable outcome of multiple pregnancies.

An important limitation of this study is that MS/MS to confirm the identification of the lipids was not performed, but this study is to be continued to confirm our findings.

In conclusion, our evidence suggests that MS fingerprinting is a useful predictive tool for blastocysts that fail to implant, and therefore this technique could be incorporated in the laboratory routine, adjunct to morphology evaluation to identify embryos that should not be transferred.

## References

[r1] ACOG (2005). ACOG Committee Opinion #324: Perinatal risks associated with
assisted reproductive technology. Obstet Gynecol.

[r2] Adashi EY, Barri PN, Berkowitz R, Braude P, Bryan E, Carr J, Cohen J, Collins J, Devroey P, Frydman R, Gardner D, Germond M, Gerris J, Gianaroli L, Hamberger L, Howles C, Jones Jr H, Lunenfeld B, Pope A, Reynolds M, Rosenwaks Z, Shieve LA, Serour GI, Shenfield F, Templeton A, van Steirteghem A, Veeck L, Wennerholm UB (2003). Infertility therapy-associated multiple pregnancies (births): an
ongoing epidemic. Reprod Biomed Online.

[r3] Ahlström A, Wikland M, Rogberg L, Barnett JS, Tucker M, Hardarson T (2011). Cross-validation and predictive value of near-infrared
spectroscopy algorithms for day-5 blastocyst transfer. Reprod Biomed Online.

[r4] Alfarawati S, Fragouli E, Colls P, Stevens J, Gutiérrez-Mateo C, Schoolcraft WB, Katz-Jaffe MG, Wells D (2011). The relationship between blastocyst morphology, chromosomal
abnormality, and embryo gender. Fertil Steril.

[r5] Assou S, Boumela I, Haouzi D, Anahory T, Dechaud H, De Vos J, Hamamah S (2011). Dynamic changes in gene expression during human early embryo
development: from fundamental aspects to clinical
applications. Hum Reprod Update.

[r6] Aydiner F, Yetkin CE, Seli E (2010). Perspectives on emerging biomarkers for non-invasive assessment
of embryo viability in assisted reproduction. Curr Mol Med.

[r7] Bligh EG, Dyer W J (1959). A rapid method of total lipid extraction and
purification. Can J Biochem Physiol.

[r8] Botros L, Sakkas D, Seli E (2008). Metabolomics and its application for non-invasive embryo
assessment in IVF. Mol Hum Reprod.

[r9] Braga DP, Setti AS, Cabral EC, Eberlin M, Lo Turco EG, Borges Jr E (2015). "Non-Invasive Prediction of Blastocyst Formation by Day Three
Embryo Culture Medium Mass Spectrometry Lipid Fingerprinting. JBRA Assist Reprod.

[r10] Bromer JG, Seli E (2008). Assessment of embryo viability in assisted reproductive
technology: shortcomings of current approaches and the emerging role of
metabolomics. Curr Opin Obstet Gynecol.

[r11] Cortezzi SS, Cabral EC, Trevisan MG, Ferreira CR, Setti AS, Braga DP, Figueira Rde C, Iaconelli Jr A, Eberlin MN, Borges Jr E (2013). Prediction of embryo implantation potential by mass spectrometry
fingerprinting of the culture medium. Reproduction.

[r12] Cortezzi SS, Garcia JS, Ferreira CR, Braga DP, Figueira RC, Iaconelli Jr A, Souza GH, Borges Jr E, Eberlin MN (2011). Secretome of the preimplantation human embryo by bottom-up
label-free proteomics. Anal Bioanal Chem.

[r13] Dominguez F, Pellicer A, Simon C (2003). The chemokine connection: hormonal and embryonic regulation at
the human maternal-embryonic interface-a review. Placenta.

[r14] ESHRE Capri Workshop Group (2014). Birth defects and congenital health risks in children conceived
through assisted reproduction technology (ART): a meeting
report. J Assist Reprod Genet.

[r15] Ferreira CR, Turco EGL, Saraiva SA, Bertolla RP, Perecin F, Souza GHFM, Murgu M, Garcia J.S, Cortezzi SS, Meirelles FV, Klitzke CF, Cabral EC, Miglino MA, Porciuncula P M, Leal CLV, Borges Jr. E, Martins D S, Ambrósio CE, D'Alexandri F, Smith LC, Eberlin MN (2010). Proteomics, Metabolomis and Lipidomics in Reproductive
Biotechnologies: The MS Solutions. Acta Sci Vet.

[r16] Grady R, Alavi N, Vale R, Khandwala M, McDonald SD (2012). Elective single embryo transfer and perinatal outcomes: a
systematic review and meta-analysis. Fertil Steril.

[r17] Hamel M, Dufort I, Robert C, Gravel C, Leveille MC, Leader A, Sirard MA (2008). Identification of differentially expressed markers in human
follicular cells associated with competent oocytes. Hum Reprod.

[r18] Han X, Gross RW (2005). Shotgun lipidomics: electrospray ionization mass spectrometric
analysis and quantitation of cellular lipidomes directly from crude extracts
of biological samples. Mass Spectrom Rev.

[r19] Katz-Jaffe MG, McReynolds S, Gardner DK, Schoolcraft WB (2009). The role of proteomics in defining the human embryonic
secretome. Mol Hum Reprod.

[r20] Kovalevsky G, Patrizio P (2005). High rates of embryo wastage with use of assisted reproductive
technology: a look at the trends between 1995 and 2001 in the United
States. Fertil Steril.

[r21] Loizides-Mangold U (2013). On the future of mass-spectrometry-based
lipidomics. FEBS J.

[r22] Mastenbroek S, van der Veen F, Aflatoonian A, Shapiro B, Bossuyt P, Repping S (2011). Embryo selection in IVF. Hum Reprod.

[r23] Oresic M, Hänninen VA, Vidal-Puig A (2008). Lipidomics: a new window to biomedical frontiers. Trends Biotechnol.

[r24] Palermo G, Joris H, Devroey P, Van Steirteghem AC (1992). Pregnancies after intracytoplasmic injection of single
spermatozoon into an oocyte. Lancet.

[r25] Pandian Z, Bhattacharya S, Ozturk O, Serour G, Templeton A (2009). Number of embryos for transfer following in-vitro fertilisation
or intra-cytoplasmic sperm injection. Cochrane Database Syst Rev.

[r26] Paternot G, Devroe J, Debrock S, D'Hooghe TM, Spiessens C (2009). Intra- and inter-observer analysis in the morphological
assessment of earlystage embryos. Reprod Biol Endocrinol.

[r27] Sakkas D, Gardner DK (2005). Noninvasive methods to assess embryo quality. Curr Opin Obstet Gynecol.

[r28] Santos-Filho E, Noble JA, Wells D (2010). A review on automatic analysis of human embryo microscope
images. Open Biomed Eng J.

[r29] Schwudke D, Oegema J, Burton L, Entchev E, Hannich JT, Ejsing CS, Kurzchalia T, Shevchenko A (2006). Lipid profiling by multiple precursor and neutral loss scanning
driven by the data-dependent acquisition. Anal Chem.

[r30] Scott R, Seli E, Miller K, Sakkas D, Scott K, Burns DH (2008). Noninvasive metabolomic profiling of human embryo culture media
using Raman spectroscopy predicts embryonic reproductive potential: a
prospective blinded pilot study. Fertil Steril.

[r31] Seli E, Botros L, Sakkas D, Burns DH (2008). Noninvasive metabolomic profiling of embryo culture media using
proton nuclear magnetic resonance correlates with reproductive potential of
embryos in women undergoing in vitro fertilization. Fertil Steril.

[r32] Seli E, Vergouw CG, Morita H, Botros L, Roos P, Lambalk CB, Yamashita N, Kato O, Sakkas D (2010). Noninvasive metabolomic profiling as an adjunct to morphology for
noninvasive embryo assessment in women undergoing single embryo
transfer. Fertil Steril.

[r33] Setti PE, Bulletti C (2011). Strategies to improve embryo implantation to supraphysiological
rates. Ann N Y Acad Sci.

[r34] Sfontouris IA, Lainas GT, Sakkas D, Zorzovilis IZ, Petsas GK, Lainas TG (2013). Non-invasive metabolomic analysis using a commercial NIR
instrument for embryo selection. J Hum Reprod Sci.

[r35] Singh R, Sinclair KD (2007). Metabolomics: approaches to assessing oocyte and embryo
quality. Theriogenology.

[r36] Steinberg ML, Boulet S, Kissin D, Warner L, Jamieson DJ (2013). Elective single embryo transfer trends and predictors of a good
perinatal outcome--United States, 1999 to 2010. Fertil Steril.

[r37] Steptoe PC, Edwards RG (1978). Birth after the reimplantation of a human embryo. Lancet.

[r38] Vergouw CG, Botros LL, Roos P, Lens JW, Schats R, Hompes PG, Burns DH, Lambalk CB (2008). Metabolomic profiling by near-infrared spectroscopy as a tool to
assess embryo viability: a novel, non-invasive method for embryo
selection. Hum Reprod.

[r39] Want EJ, Cravatt BF, Siuzdak G (2005). he expanding role of mass spectrometry in metabolite profiling
and characterization. Chembiochem.

